# Efficiency and Cost of Economical Brain Functional Networks

**DOI:** 10.1371/journal.pcbi.0030017

**Published:** 2007-02-02

**Authors:** Sophie Achard, Ed Bullmore

**Affiliations:** Brain Mapping Unit, Department of Psychiatry, Addenbrooke's Hospital, University of Cambridge, Cambridge, United Kingdom; University College London, United Kingdom

## Abstract

Brain anatomical networks are sparse, complex, and have economical small-world properties. We investigated the efficiency and cost of human brain functional networks measured using functional magnetic resonance imaging (fMRI) in a factorial design: two groups of healthy old (*N* = 11; mean age = 66.5 years) and healthy young (*N* = 15; mean age = 24.7 years) volunteers were each scanned twice in a no-task or “resting” state following placebo or a single dose of a dopamine receptor antagonist (sulpiride 400 mg). Functional connectivity between 90 cortical and subcortical regions was estimated by wavelet correlation analysis, in the frequency interval 0.06–0.11 Hz, and thresholded to construct undirected graphs. These brain functional networks were small-world and economical in the sense of providing high global and local efficiency of parallel information processing for low connection cost. Efficiency was reduced disproportionately to cost in older people, and the detrimental effects of age on efficiency were localised to frontal and temporal cortical and subcortical regions. Dopamine antagonism also impaired global and local efficiency of the network, but this effect was differentially localised and did not interact with the effect of age. Brain functional networks have economical small-world properties—supporting efficient parallel information transfer at relatively low cost—which are differently impaired by normal aging and pharmacological blockade of dopamine transmission.

## Author Summary

It is increasingly evident that many complex networks, in diverse fields and over a wide range of spatial and time scales, may have topological properties in common. These unifying organizational principles have been described in terms of “small-world” parameters—meaning that many networks have both local clustering of connections and a short path length between any pair of nodes. Recent work has shown that we can also define small-world networks as having high global and local efficiency of parallel information transfer; and that many networks are economical in the sense of supporting high efficiency for low cost. Here we extend these ideas for the first time to an analysis of human brain functional networks derived from functional magnetic resonance imaging data. We show that human brain functional networks have economical small-world properties and that economical performance of these networks is detrimentally but differently affected by normal aging and by treatment with a dopamine receptor antagonist. The results illustrate concepts and techniques that could be important in further exploration of developmental, pathological, and pharmacological effects on human brain functional networks. They are also likely to be generalisable to applications in other fields of computational biology.

## Introduction

### Small-World Brain Networks

Complex networks, from ecosystems to metabolic pathways, occur in diverse fields of biological science [1,2]. Nervous systems are complex networks at multiple scales of time and space. It has been shown that brain anatomical and functional networks are topologically intermediate between highly regular lattices and random graphs [3,4]. In other words, brain networks have characteristically small-world properties of dense or clustered local connectivity with relatively few long-range connections mediating a short path length between any pair of neurons or regions in the network [5–7]. Small-world topology is an attractive model for brain network organization because it could support both segregated and distributed information processing [8], confer resilience against pathological attack [9], and minimise wiring costs [10]. From an evolutionary perspective, it can be argued that small-world brain networks have been competitively selected to solve the economic problem of maximising information processing efficiency while minimising costs [6,7,11].

### Hypotheses and Study Design

To test directly the hypothesis that small-world brain functional networks have economical properties, we measured global and local efficiency of parallel information processing, as a function of cost, in networks derived from functional magnetic resonance imaging (fMRI) data. Economical network organization has been previously defined in terms of high efficiency *E* on the basis of low cost *K* [12,13]. Efficiency was defined as a function of the minimum path length between regions; the most simple measure of cost was defined as the number of edges or connections in the network; both metrics were expressed in proportion to the maximum efficiency and cost of a comparable network comprising all possible connections between regions [12,13].

We predicted that brain functional networks would generally demonstrate economical small-world properties of high global and local efficiency for low cost. The functional connectivity between each possible pair of *N* = 90 regional fMRI time series was first estimated in the low-frequency interval 0.06–0.11 Hz using wavelet correlation analysis [9]. The resulting wavelet correlation matrices (one for each participant in the study) were then thresholded to produce a set of undirected graphs representing the brain functional network for each participant, and the efficiency of each network was measured as a function of variable cost in the range 0.01 ≤ *K* ≤ 0.5, which is equivalent to 1%–50% of all possible edges in a (fully connected) network of *N* = 90 nodes.

We were also interested to test the subsidiary hypothesis that the economical performance of small-world brain functional networks would be adversely affected by normal aging and by pharmacological blockade of dopamine neurotransmission. We therefore acquired and analysed fMRI data from two age-defined groups of healthy volunteers—a young group (*N* = 15; mean age = 24.7 years) and an older group (*N* = 11; mean age = 66.5 years)—each scanned in a no-task or resting state on two separate occasions following oral administration either of placebo or of sulpiride 400 mg, a selective dopamine D2 receptor antagonist.

### Methodological Motivations

Given the very large number of ways in which a dataset of this complexity could be analysed, it is reasonable to ask how this particular sequence of operations—wavelet correlation analysis followed by thresholding and economical small-world analysis of the resulting graphs—was motivated a priori.

We used *wavelets* for estimation of functional connectivity in these data because of prior evidence indicating that cortical fMRI time series often have long memory in time (slowly decaying positive autocorrelation), or a 1/*f* power spectrum in the frequency domain [14,15]. This property argues against measuring functional connectivity between a pair of fMRI time series by estimation of their covariance or correlation in the time domain, or their coherence in the frequency domain, because both time- and frequency-domain estimators of association are not properly estimable for long memory processes [16]. The wavelet domain, on the other hand, is technically advantageous for analysis of long memory and potentially nonstationary fMRI time series [17]. For example, the wavelet coefficients of a long memory process are stationary, and wavelet-based estimators of the correlation between two long memory processes have been shown to have desirable statistical properties [18,19]. An additional advantage of wavelet-based connectivity analysis, shared in common with approaches in the frequency domain, is that it allows us to focus on the functional association between brain regions subtended by activity in a defined frequency interval or wavelet scale. This is attractive for an application to resting-state fMRI data because there is prior evidence indicating that functional connectivity between regions is typically greatest at frequencies less than 0.1 Hz [20–23], whereas non-neural sources of resting state correlations, such as aliased cardiorespiratory effects, are subtended by higher frequencies [22,24]. (We return in the Discussion section to the question of what oscillations or correlations at these so-called infraslow frequencies might represent.)

The rationale for *thresholding* was simply that we wished to focus attention on the topology of relatively sparse (low-cost) networks, representing the strongest functional connections or largest wavelet correlations. This is broadly justified by the known sparsity of anatomical connections in non-human and human nervous systems; in *Caenorhabditis elegans,* for example, less than 6% of all possible unweighted synaptic connections exist between neurons [13]. There are, of course, methods for analysis of functional connectivity matrices that do not demand preliminary thresholding; one could perhaps apply principal component analysis to the wavelet correlation matrix to produce frequency-specific eigenimages. However, here we were interested primarily in the topological properties of networks or graphs—and thresholding is a necessary step in the derivation of graphs, whether weighted or unweighted, from any continuous measure of association between variables. The choice of threshold then becomes a critical issue which we addressed by estimating and integrating network efficiency over a range of correlation thresholds equivalent to costs in the range 0.01 ≤ *K* ≤ 0.5 (see Results).

The main motivation for our adoption of the relatively novel *metrics of network efficiency*, first defined by Latora and Marchiori [12,13], was our hypothetical interest in directly estimating the economical performance of small-world brain functional networks. We note that global and local efficiency have previously been estimated for anatomical networks in macaque and cat cortex [12] (see Table 1); however, there has been no previous application of these economical small-world metrics to human brain functional network analysis. Global efficiency (*E_global_,* Equation 1) is inversely related to the “classical” small-world metric of average minimum path length, *L* [5]. Thus, a small-world network will have *E_global_* greater than (*L* less than) a regular lattice but *E_global_* less than (*L* greater than) a random network. This strong inverse relation might suggest that the two metrics are of equal merit in any application; however, efficiency offers a number of technical and conceptual advantages. First, average minimum path length was defined only for connected graphs, i.e., graphs in which all nodes are connected as part of one giant cluster or large subgraph, yet application of high thresholds to correlation matrices may result in one or more of the less strongly connected regions becoming disconnected from the giant cluster. In this case, the path length between the disconnected region and the rest of the network will be infinite, whereas the contribution of the disconnected region to the global efficiency of the network will be zero. Thus, inverting path length in the calculation of global efficiency simplifies the numerical issues in estimating efficiency of unconnected regions. More generally, we can see that inversion of path length also has the intuitively desirable effect of weighting global efficiency by the contribution of the most highly connected regions or “hubs,” whereas average minimum path length is weighted by the least connected regions or “diaspora.”

**Table 1:**
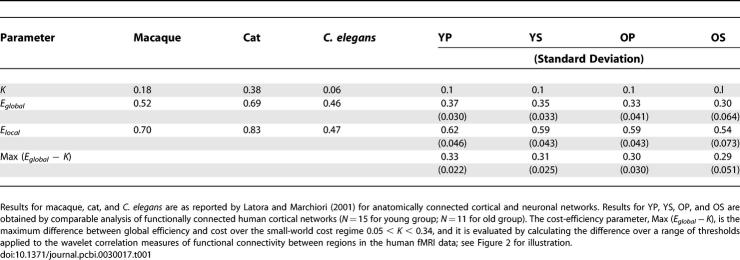
Efficiency *E* and Cost *K* Parameters for Brain Networks

Second, average minimum path length was defined only for unweighted graphs (in which the connections or edges between nodes are all assigned equal value 1); whereas the efficiency metrics are applicable both to unweighted and weighted graphs (in which the edges are assigned different values depending on the continuously variable strength of functional association or the physical distance between nodes). Here we will focus mostly on the simpler case of unweighted graph analysis but we also rehearse the formulation of efficiency metrics for weighted graphs and report some additional results for analysis of brain networks weighted by the strength of functional association between regions. Third, path length provides a measure of the network's capacity for serial information transfer between nodes, whereas global efficiency is a measure of the network's capacity for parallel information transfer between nodes via multiple series of edges. Since there is strong prior evidence that the brain supports massively parallel information processing, it seems conceptually preferable to adopt efficiency metrics of brain functional network topology.

**Figure 1:**
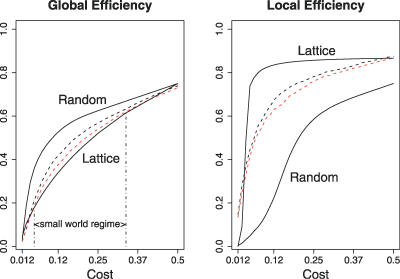
Small-World Properties of Human Brain Functional Networks Global and local efficiency (*y*-axis) as a function of cost (*x*-axis) for a random graph, a regular lattice, and brain networks. For all networks, global and local efficiency increase with cost; the random graph has greater global efficiency than the lattice; the lattice has greater local efficiency than the random graph. On average, over all subjects in each group, young brain networks (black broken lines) and old brain networks (red broken lines) have efficiency curves located between the limiting cases of random and lattice topology. The small-world regime is conservatively defined as the range of costs 0.34 ≤ *K* ≤ 0.5 for which the global efficiency curve for the old networks is greater than the global efficiency curve for the lattice.

## Results

### Brain Functional Networks Have Economical Small-World Properties

Global and local efficiency of brain networks were compared with the same parameters estimated in a random graph and a regular lattice over a range of network costs. As expected, efficiency monotonically increased as a function of cost in all networks; the random graph had higher global efficiency than the lattice; and the lattice had higher local efficiency than the random graph, for costs in the range 0 ≤ *K* ≤ 0.5; see Figure 1.

The efficiency curves of the brain networks were intermediate between these limiting cases: brain networks had global efficiency greater than the lattice but less than the random graph, and local efficiency greater than random but less than lattice. This characteristically small-world behaviour of the brain networks was most consistently seen for low-cost to medium-cost networks, 0.05 ≤ *K* ≤ 0.34, comprising between 5% and 34% of the 4,005 possible edges in a network of 90 nodes.

**Figure 2:**
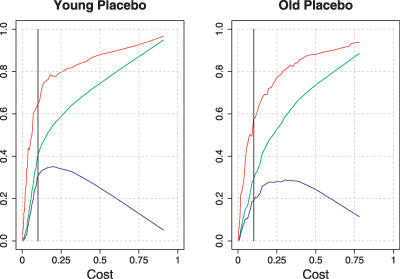
Economical Properties of Human Brain Functional Networks Efficiency (*y*-axis) as a function of cost (*x*-axis) for two individual brain networks: a young subject following placebo (left panel) and an older subject following placebo (right panel). For both subjects, local efficiency (red lines) and global efficiency (green lines) increase monotonically with cost; and cost efficiency (the difference between global efficiency and cost; blue lines) has its maximum value when 0.2 ≤ *K* ≤ 0.4. The low-cost threshold, *K* ∼ 0.1, associated with the sparse networks reported in Figures 3 and 5, is shown as a black vertical line in each graph.

Importantly, in this small-world cost regime, 0.05 ≤ *K* ≤ 0.34, global and local efficiency of brain networks was greater than cost. The difference between global efficiency and cost—called cost efficiency—typically had a maximum positive value in networks comprising about 850 edges or 21% of the possible edges in the network, i.e., Max(*E_global_* − *K*) when *K* ∼ 0.2; see Figure 2.

### Age Impairs Economical Performance of Small-World Brain Networks

**Figure 3:**
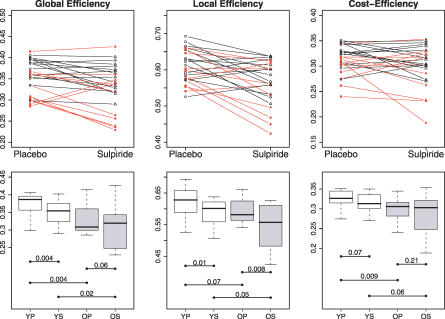
Efficiency Measures for Low-Cost Brain Functional Networks, with *K* ∼ 0.1, in All Participants (Top row) Within-subject effects of dopamine antagonism (sulpiride 400 mg) on global efficiency (left column), local efficiency (middle column), and maximum cost efficiency (right column): data on younger subjects are denoted by black lines and black triangles, data on older subjects by red lines and red triangles. (Bottom row) Box-plots showing median, interquartile range, and range for global efficiency, local efficiency, and maximum-cost efficiency in each age group after each treatment. Each horizontal line and the associated number represent the *p*-value of a post-hoc *t*-test: there were consistent pairwise differences in global efficiency related to age (YP–OP and YS–OS), less consistent age-related differences in cost efficiency (YP–OP), and no significant post-hoc effects of age on local efficiency; however, there were consistent pairwise differences in local efficiency related to drug treatment (YP–YS and OP–OS), and a significant effect of drug on global efficiency in young people (YP–YS).

Older people had reduced global and local efficiency of brain functional networks compared with younger people, as shown by the summary statistics on economical parameters of sparse networks with *K* ∼ 0.1 (Table 1 and Figure 3) or by inspection of the individual network efficiency estimates (Figure 3). This detrimental effect of age on global efficiency was statistically significant (ANOVA [analysis of variance], *F* = 8.96, df = 1,24, *p* = 0.006), as was the effect of age on local efficiency (ANOVA, *F* = 4.41, df = 1,24, *p* = 0.05). Results of post-hoc *t*-tests are also shown in Figure 3.

**Figure 4:**
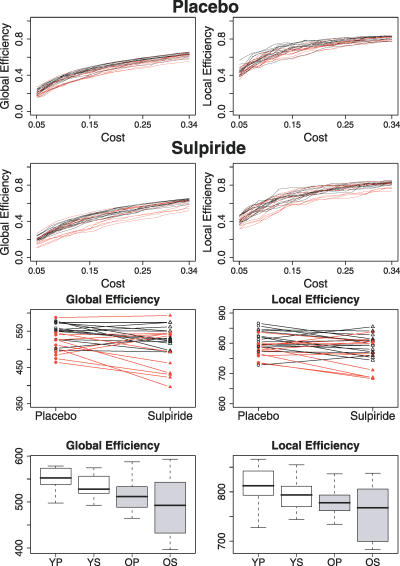
Efficiency Measures Estimated and Integrated over the Small-World Regime of Network Costs, 0.05 ≤ *K* ≤ 0.34 (Rows 1,2) Global (left) and local (right) efficiency curves for young (black lines) and older (red lines) people after placebo (row 1) and sulpiride (row 2). (Row 3) Within-subject effects of dopamine antagonism on integrated global efficiency (left) and integrated local efficiency (right): data on younger subjects are denoted by black lines and black triangles, data on older subjects by red lines and red triangles. (Row 4) Box-plots showing median, interquartile range, and range for integrated global efficiency (left) and integrated local efficiency (right) in each age group after each treatment.

Age-related differences in efficiency were not unique to the single threshold used to generate low-cost networks with *K* ∼ 0.1. As shown in Figure 4, older people had reduced global and local efficiency over the entire range of network costs comprising the small-world regime, 0.05 ≤ *K* ≤ 0.34. The integrals of the global and local efficiency curves were used as summary measures of network efficiency over the small-world regime, and analysis of variance confirmed detrimental effects of age on integrated global efficiency (*F* = 8.47, df = 1,24, *p* = 0.008) and integrated local efficiency (*F* = 6.19, df = 1,24, *p* = 0.02).

Age also significantly reduced the maximum-cost efficiency (ANOVA, *F* = 6.82, df = 1,24, *p* = 0.015); see Table 1 and Figure 3.

### Dopamine Blockade Impairs Economical Performance of Small-World Brain Networks

A single dose of sulpiride, compared with placebo, reduced global and local efficiency of low-cost networks, with *K* ∼ 0.1, in both young and old people, although drug effects were more variable in the older age group (see Table 1 and Figure 3). The detrimental effect of drug on global efficiency was significant (ANOVA, *F* = 9.43, df = 1,24, *p* = 0.005), as was its effect on local efficiency (ANOVA, *F* = 14.36, df = 1,24, *p* = 0.0009). Results of post-hoc *t*-tests are also shown in Figure 3. There were no significant age by drug interactions on global or local efficiency.

Drug-related effects on efficiency were evident not only in the low-cost network with *K* ∼ 0.1 but also in terms of global and local efficiency curves integrated over the small-world cost regime, 0.05 ≤ *K* ≤ 0.34 (Figure 4). The detrimental effect of sulpiride on integrated global efficiency was significant (ANOVA, *F* = 5.40, df = 1,24, *p* = 0.03), as was its effect on integrated local efficiency (ANOVA, *F* = 6.11, df = 1,24, *p* = 0.02). There were no significant age by drug interactions on integrated global or local efficiency.

There was no significant effect of drug on maximum-cost efficiency.

### Age and Drug Effects on Network Efficiency Are Differentially Localised

**Figure 5:**
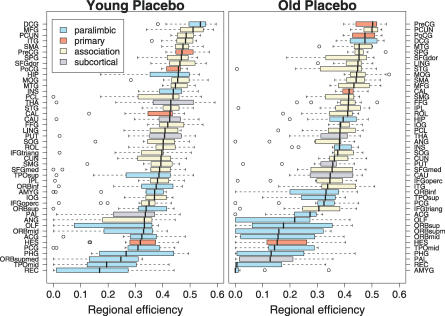
Effect of Age on Regional Efficiency Brain regions ranked in order of decreasing regional efficiency: “hubs” have high regional efficiency and tend to be larger regions of association cortex in both young (left panel) and old people (right panel); “diaspora” have low regional efficiency and tend to be smaller regions of limbic/paralimbic cortex. Each box-plot shows median, interquartile range, and range for individual estimates of regional efficiency in each age group following placebo; boxes are color-coded to differentiate primary sensory or motor cortex (red), unimodal or multimodal association cortex (beige), limbic or paralimbic cortex (turquoise blue), and subcortical nuclei (periwinkle blue).

The regional efficiency of each node is a measure of its connectivity to all other nodes of the network; highly connected nodes or “hubs” will have high regional efficiency. When the 90 brain regional nodes were ranked in order of decreasing regional efficiency (using data acquired in young people following placebo [YP]), we found that the hubs of the network were predominantly larger regions of unimodal or multimodal association cortex, whereas smaller limbic/paralimbic regions often had relatively low regional efficiency; see Figure 5. A broadly similar pattern was found in data acquired from older subjects following placebo (Figure 5), indicating that association cortical areas tend to be hubs of the brain functional network regardless of age (and treatment with sulpiride; unpublished data).

**Figure 6:**
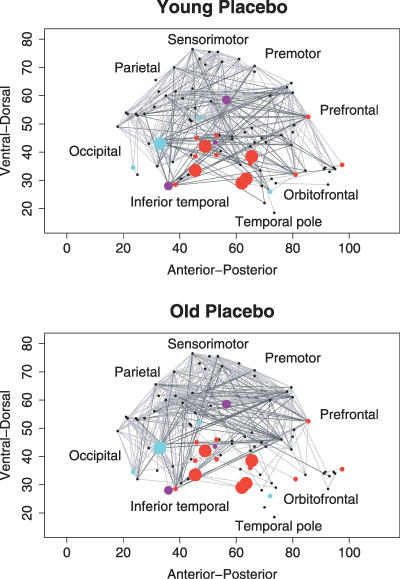
Anatomical Representation of Brain Functional Networks Highlighting Regional Effects of Age and Dopamine Receptor Antagonism Brain functional networks for one young person following placebo (top row) and one old person following placebo (bottom row). These networks were constructed by thresholding the individual wavelet correlation matrices to derive sparse networks with equal cost, *K* ∼ 0.1: regional nodes are shown as dots or circles in a sagittal view of the right side of the brain; strong functional connections are shown as undirected edges between nodes. The size of regional nodes is proportional to the significance of age-related or drug-related reductions in regional efficiency: red nodes, efficiency reduced by older age; blue nodes, efficiency reduced by sulpiride; purple nodes, efficiency reduced by both older age and sulpiride. See Table 2 for anatomical detail concerning locations of significant difference in regional efficiency.

However, multiple univariate analysis of variance showed that older age was associated with significantly reduced nodal efficiency of several regions of frontal and temporal neocortex; paralimbic and limbic regions including hippocampus, parahippocampal gyrus, amygdala, and dorsal cingulate gyrus; and subcortical regions including pallidum and thalamus; see Table 2 for details and Figure 6 for a network map of brain regions with significantly reduced nodal efficiency due to older age. The less extensive effects of dopamine blockade on regional efficiency were most clearly evident in temporal neocortex and dorsal cingulate gyrus; Table 2 and Figure 6.

**Table 2:**
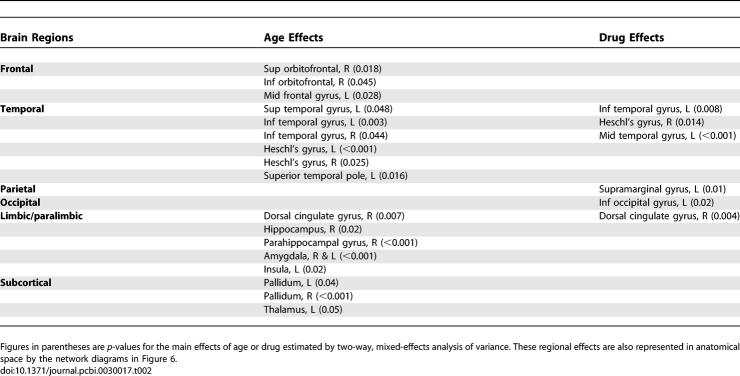
Significant Effects of Age and Drug Treatment on Nodal Efficiency of Regions in a Low-Cost Brain Functional Network with *K* ∼ 0.1

## Discussion

### Economical Models of Brain Function

It is well-recognised that the metabolic costs of building and functionally resourcing the brain are large in proportion to the total energy budget of the body [25]. There is evidence that many aspects of brain anatomy can be explained in terms of minimising axonal wiring or metabolic running costs [26,27]. It therefore seems likely that one of the selection pressures driving brain evolution has been minimisation of costs—favoring a high density of short-range local connections. However, it also seems likely that brains have evolved to support greater diversity and adaptivity of sensorimotor and cognitive functions, presumably by enhancing the neuronal infrastructure for information processing—and this would be expected to favor selection of a few long-range connections mediating efficient information transfer between spatially distributed regions [7,11]. We can surmise that, broadly speaking, brains might have been competitively selected to maximise *cost efficiency* of parallel information processing in large-scale networks.

There is data in support of this hypothesis from computational modeling studies by Sporns et al. [28], which showed that graphs computationally evolved for high complexity of dynamic behaviour had a sparse, small-world topology of edges between connected nodes. However, there have been no previous efforts directly to measure economical parameters of brain networks derived empirically from human functional neuroimaging data. Here, we have shown that human brain functional networks have topological properties that would be expected on the hypothesis of economic selection: high efficiency of parallel information transfer at low cost, e.g., about 35% of maximum efficiency for 10% of maximum cost (Table 1).

### Effects of Age and Dopamine Blockade on Economical Network Parameters

We have also shown that the economical small-world properties of these networks are deleteriously affected by both older age and blockade of dopamine D2 receptors.

In both age groups, the hubs of the functional networks were predominantly larger regions of multimodal and unimodal association cortex. This observation replicates prior studies in independent samples [8,9], implicating recently evolved association cortical regions as critical nodes in monkey and human brain anatomical and functional networks. The deleterious effects of older age on network performance could be localized in terms of reduced regional efficiency of hubs such as dorsal cingulate, middle frontal, and inferior temporal gyri. However, there was also clear evidence for age-related deficits in efficiency of topologically more peripheral limbic and paralimbic regions, notably hippocampus, parahippocampal gyrus, and amygdala.

Pharmacological blockade of dopamine D2 receptors by a single dose of sulpiride 400 mg was also acutely associated with impaired global and local efficiency of the networks. These effects were less salient and extensive than the effects of age, and in the older group there was considerable between-subject variability in drug effects. Dopamine transmission is known to play an important role in modulating the frequency, phase, and spatial coherence of endogenous oscillations in the basal ganglia and cortex [29–33]. Our data suggest more specifically that dopaminergic afferentation may normally contribute to optimising the economical performance of small-world brain functional networks, and pharmacological antagonism of this modulatory input impairs network efficiency by an effect most clearly localised to dorsal cingulate and lateral temporal cortical hubs.

Since increasing age has been consistently associated with loss of dopamine cell bodies and decreased density of dopamine projections to striatum and cortex, contributing to age-related cognitive decline [34–36], it is conceivable that the effects of age on economical network performance might be mimicked by the effects of dopamine receptor antagonism. This was true in the broad sense that the D2 receptor blockade, like older age, reduced global and local network efficiency: both factors degraded the relatively optimal network performance demonstrated by young people following placebo. More locally, both age and sulpiride impaired regional efficiency of dorsal cingulate gyrus, which is known to receive a major dopaminergic input and to be vulnerable to regressive changes in normal aging. However, the effects of age were generally more salient and extensive, involving several frontal and limbic/paralimbic regions that were not significantly affected by sulpiride. We conclude that attenuated dopamine transmission may contribute to age-related impairments in functional network efficiency, but it seems likely that additional mechanisms must be invoked to account for the topological marginalisation of medial temporal and frontal regions in older people. For example, this functional observation might be related to structural neuroimaging evidence for grey and white matter deficits of frontal and temporal regions due to normal aging [37,38].

### Low-Frequency Oscillations and Correlations in fMRI

Many previous studies of fMRI data acquired in a no-task or “resting” state have demonstrated significant inter-regional correlations subtended by very-low-frequency oscillations, less than 0.1 Hz [4,20–22,39]. Various possible causes have been considered for these observations including instrumental, vasomotor [40], and neuronal mechanisms. fMRI studies using rapid sampling rates, i.e., short repetition times (TR), have shown that low-frequency power or coherence is unlikely to represent (possibly aliased) signals related to the cardiac or respiratory cycles [22,24]. On the other hand, multisecond periodicities or infraslow oscillations have been reported in both local field potential recordings from rat basal ganglia and monkey cortex [31,41,42] and the surface EEG in humans [43]. These electrophysiological data, unconfounded by the vascular or systemic cardiorespiratory effects that can complicate interpretation of blood-oxygen-level-dependent (BOLD) signals, suggest that coherent neuronal oscillations exist at very low frequencies and could represent a plausible substrate for the low-frequency oscillations and correlations observed using fMRI. The cognitive implications of these so-called “resting state networks,” and their relationships to large-scale brain systems coherently oscillating at higher frequencies [44], remain to be elucidated. One important limitation of the existing fMRI literature on low-frequency connectivity is that it has focused predominantly on data acquired in resting or no-task states, which are experimentally uncontrolled and are not well-designed to clarify the significance of low-frequency correlations for cognitive function.

An additional caveat in interpretation of these results is that dopaminergic drugs may have effects on BOLD signals that are mediated by dopamine receptors on the small arterial walls of the cerebral vasculature [45] rather than by neuronal receptors (see Honey and Bullmore [46] for a review of issues in pharmacological studies using fMRI). This interpretation of our fMRI data is difficult to rule out completely without access to complementary electrophysiological data showing similar effects of sulpiride on low-frequency brain networks. However, we have previously reported that the effects of sulpiride on parameters of human cortical response to somatosensory stimulation were similar whether measured by fMRI or by evoked potentials, implying that measurements of sulpiride's effects on BOLD signal change can indeed reflect an underlying change in neuronal activity [47].

## Materials and Methods

### Sample.

Thirty healthy human volunteers were recruited in two age groups: 17 younger participants aged 18–33 years, mean age = 24.3 years, nine male; and 13 older participants aged 62–76 years, mean age = 67.3 years, six male. Recruitment was via locally placed advertisements, followed by telephone screening using a standard questionnaire. Exclusion criteria included a history of neurological or psychiatric disorder, current treatment with vasoactive or psychotropic medication, or any contraindications to MRI or study drug. Prior to functional MRI scanning, each participant also had an electrocardiogram and a structural MRI scan reviewed as normal by a physician.

The two age groups were matched on education and on crystallised IQ, as estimated by the National Adult Reading Test [48]. Older volunteers also completed the Mini Mental State Examination, and a minimum score of 28 was required for participation [49,50].

All participants gave informed consent in writing. The study was approved by the Addenbrooke's National Health Service Trust Local Research Ethics Committee, Cambridge, UK.

### Experimental design.

We used a double-blind placebo-controlled crossover design, each participant attending for two study sessions separated by at least one week. In one session, subjects received an oral placebo; in the other session, subjects received a single oral dose of sulpiride 400 mg, a selective dopamine D2 receptor antagonist. The order of treatments was randomized, and both treatments were administered four hours before functional MRI. Prior pharmacokinetic data have shown that the time to maximum plasma concentration of sulpiride is three hours after an oral dose, and plasma levels remain close to maximum until six hours after dosing.

### Functional MRI data acquisition and pre-processing.

Each participant was scanned lying quietly at rest with eyes closed for 9 min, 37.5 s. Gradient-echo echoplanar imaging (EPI) data depicting BOLD contrast were acquired using a Medspec S300 scanner (Bruker Medical, http://bruker-medical.de) operating at 3.0 T in the Wolfson Brain Imaging Centre (Cambridge, UK). We acquired 525 volumes with the following parameters: number of slices, 21 (interleaved); slice thickness, 4 mm; interslice gap, 1 mm; matrix size, 64 × 64; flip angle, 90°; repetition time (TR), 1100 ms; echo time, 27.5 ms; in-plane resolution, 3.125 mm. The first seven volumes were discarded to allow for T1 saturation effects, leaving 518 volumes available for analysis of resting state connectivity in each subject.

Each dataset was corrected initially for geometric displacements because of head movement and co-registered with the Montreal Neurological Institute gradient-echo echoplanar imaging (EPI) template image, using an affine transform implemented in SPM2 software (http://www.fil.ion.ucl.ac.uk). Four datasets (two young, two old) that had been affected by head movement in excess of 3 mm translation, or 0.3° rotation, in *x, y,* or *z* dimensions, were discarded. The remaining data were not spatially smoothed before regional parcellation using the anatomically labeled template image validated previously by Tzourio-Mazoyer et al. [51]. This parcellation divides each cerebral hemisphere into 45 anatomical regions of interest. Regional mean time series were estimated for each individual by averaging the fMRI time series over all voxels in each of 90 regions. Each regional mean time series was further corrected for the effects of head movement by regression on the time series of translations and rotations of the head estimated in the course of initial movement correction by image realignment. The residuals of these regressions constituted the set of regional mean time series used for wavelet correlation analysis.

### Wavelet correlation analysis and graph construction.

Wavelet transforms effect a time-scale decomposition that partitions the total energy of a signal over a set of compactly supported basis functions, or little waves, each of which is uniquely scaled in frequency and located in time. Wavelet analysis is naturally well-suited to analysis of fractal signals that have long memory or 1/*f* properties, as is typical of cortical fMRI time series in the resting state [14]; see Bullmore et al. [17] for a review of wavelet methods for fMRI data analysis. Beran [16] and Percival and Walden [52] are excellent general texts concerning long memory processes and wavelet analysis of time series, respectively.

Here we adopted a wavelet transform as a basis to estimate the scale-dependent correlations between each of 4,005 possible pairs of the *N* = 90 cortical and subcortical fMRI time series extracted from each individual dataset. We first used the maximum overlap discrete wavelet transform (MODWT) to decompose each regional mean time series into wavelet coefficients at four scales [52]. Then we estimated the pairwise inter-regional correlations between wavelet coefficients at each scale. This resulted in a set of four {90 × 90} scale-specific wavelet correlation matrices *W_m_* for each of *m* = 1,2,3,…*M* subjects [18]. Because of prior data indicating that inter-regional correlations in resting state fMRI data are particularly salient at frequencies below 0.1 Hz [9,20–22], we restricted our attention to the scale 3 wavelet-correlation matrices which represented functional connectivity in the frequency interval 0.06–0.11 Hz.

To analyze graphical properties of brain functional networks, each wavelet correlation matrix *W_m_* must be thresholded to create an adjacency matrix *A_m_,* the *a_i_*_,*j*_ elements of which are either 1, if the absolute value of the wavelet correlation between nodes *i* and *j*, *w_i_*_,*j*_ *,* exceeds a threshold value *R,* or 0, if it does not. Each adjacency matrix defines an unweighted graph *G* comprising *N* = 90 regional nodes connected by **K** undirected edges corresponding to its nonzero elements, *a_i_*_,*j*_ ≠ 0. We define the degree of each node, *k_i_, i* = 1,2,3,…*N*, as the number of edges connecting it to the rest of the graph. We also define the subgraph *G_i_* as the set of nodes that are nearest-neighbours of the *i*th node, i.e., the nodes in *G_i_* are each directly connected by a single edge to the index node, and by definition, the node *i* does not belong to the subgraph *G_i_*. The total number of edges in a graph **K** *,* divided by the maximum possible number of edges (*N* ^2^ − *N*)/2, is a simple estimator of network cost *K* [13].

Clearly the choice of threshold value 0 ≤ *R* ≤ 1 will have a major effect on the topology of the resulting networks: conservative thresholds, R → 1, will generate sparsely connected graphs (with small number of edges **K** *,* low-cost *K*); more lenient thresholds, R → 0, will generate more densely connected graphs (with large number of edges **K** *,* high-cost *K*), inevitably including a number of edges representing spurious or statistically nonsignificant correlations between regions. We adopted two complementary approaches to the choice of threshold. First, we thresholded all matrices using a single, conservative threshold chosen to construct a sparse graph with mean degree 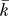 = 2log(*N*) = 9 and total number of edges **K** = 405, equivalent to *K* ∼ 0.1 or 10% of the maximum number of edges possible (4,005) in a network of 90 nodes. This allowed us to minimise the number of spurious edges in each network, to compare low-cost networks between subjects while maintaining an equivalent number of edges in each network, and to compare the topological properties of brain networks to those of equally sized random networks which require 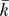 > log(*N*) to be estimable. However, any single threshold admits the criticism that the topological properties of the resulting graph may be uniquely dependent on the precise threshold value. We therefore also thresholded each matrix repeatedly over a range of costs and estimated the efficiency of the resulting graphs at each threshold value. This allowed us to compare network efficiency as a function of cost between age groups and treatment conditions in a way that was relatively independent of the precise choice of threshold. As shown in Figure S1, brain networks were not fully connected over the entire range of network costs, but the size of the giant connected cluster or largest subgraph was generally greater than 80% of the total number of regions in the network.

All data analysis and visualization was implemented in open-source, R-based software “brainwaver,” freely downloadable at http://www.wbic.cam.ac.uk/∼sa428/brainwaver.

### Efficiency and cost of small-world brain networks.

Small-world networks were originally defined by Watts and Strogatz [5] as having characteristic path length *L* less than a regular lattice and local clustering *C* greater than a random graph. It has since been shown that the inverse of the harmonic mean of the minimum path length between each pair of nodes, *L_i_*_,*j*_ *,* is a measure of the *global* efficiency of parallel information transfer *E_global_* in the network [12,13]:

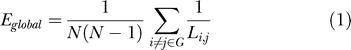
We can likewise define the *nodal* (or regional) efficiency as the inverse of the harmonic mean of the minimum path length between an index node, *i,* and all other nodes in the network:

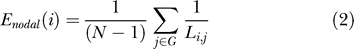
Furthermore, the clustering coefficient introduced by Watts and Strogatz [5] can be regarded as a measure of the *local* efficiency of information transfer in the immediate neighbourhood of each node [12,13]:

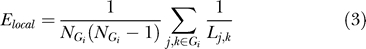
where *N_G_i__* is the number of nodes in the subgraph *G_i_*. The local efficiencies for each node can be averaged over all nodes to estimate the mean local efficiency of the graph. Since the *i*th (index) node is not an element of the subgraph *G_i_,* by definition the local efficiency can also be understood as a measure of the fault tolerance of the network, indicating how well each subgraph exchanges information when the index node is eliminated.

Global and local efficiency of brain functional networks can be compared with the same parameters estimated in random graphs and regular lattices with the same number of edges and nodes. Small-world properties of the brain networks are diagnosed by global efficiency greater than a comparable lattice (but less than a random graph) and local efficiency greater than a random graph (but less than a lattice); see Figure 1.

Global and local efficiency, and the number of edges **K** *,* for a given network can be normalised by dividing these quantities by the maximum efficiencies and number of edges that would be observed in the ideal case that the graph included all possible *N*(*N* − 1)/2 edges. By this normalization, we have 0 ≤ {*E_global_*,*E_local_*,*K*} ≤ 1. Here we define the cost efficiency as the difference between global efficiency and cost, *E_global_* − *K,* which will be positive in the case of an economical network.

### Weighted brain functional networks.

The computation of efficiency and cost can be generalised to weighted graphs. In this case, we require a weighting matrix *D* (same dimension, *N* × *N,* as the corresponding adjacency matrix *A* of the graph), whose *d_ij_* elements represent additional information about the cost of the edges. Dijkstra's algorithm can be used to search the weighted edge matrix to find the shortest weighted path length *l_i_*_,*j*_ between each pair of nodes in the graph, i.e., the minimum value of the sum of weights over all possible paths between nodes *i* and *j* [13]. The efficiency of communication between nodes *i* and *j* is then measured by global, regional, and local efficiency of the weighted graph, simply derived from Equations 1–3 by substituting *L_i_*_,*j*_ with *l_i_*_,*j*_. The cost of the weighted graph is defined by the sum of the weights between the connected nodes, *K* = ∑*_i_*_≠*j*∈*G*_ *d_i_*_,*j*_. As for unweighted graph analysis, efficiency and cost parameters can be normalised with respect to the maximum values observed when all the nodes of the graph are connected.

The main concern in modeling weighted networks is the choice of a weighting matrix. For a spatially embedded network like the brain it would generally be appropriate to use some measure of physical distance between nodes as a weighting factor. However, the length of axonal tracts between human brain regions is not yet well-known. An alternative weighting factor, more easily estimated, is a measure of the functional distance between connected regions, e.g., *d_i_*_,*j*_ = 1 − *w_i_*_,*j*_ *,* where *w_i_*_,*j*_ is the wavelet correlation coefficient for regions *i* and *j*. See Figure S2 for an analysis of functionally weighted networks that is directly comparable to the analysis of unweighted networks reported in Figure 4 on the basis of the same experimental data. It can be seen that the pattern of results for unweighted and functionally weighted networks is very similar in these data.

#### Comparison of network parameters between age and treatment groups.

To assess the significance of group differences in unweighted network topology, we used a two-way mixed-effects ANOVA model comprising age (young, old) as a between-subject factor, drug (placebo, sulpiride) as a within-subject factor, and the age by drug interaction. This model was separately fitted to each of the following dependent variables: global and local efficiency of sparsely thresholded networks with *K* ∼ 0.1; global and local efficiency curves integrated over the small-world regime of costs, 0.05 ≤ *K* ≤ 0.34; maximum-cost efficiency; and nodal efficiency for each region in the individually estimated brain functional networks. If main effects were significant by ANOVA, post-hoc *t*-tests were also conducted for the following comparisons: young people after placebo (YP) versus old people after placebo (OP), young people after sulpiride (YS) versus old people after sulpiride (OS); YP versus YS; OP versus OS.

Note that the results of the multiple ANOVAs entailed for a region-by-region analysis of nodal efficiency were regarded as significant if *p* < 0.05, i.e., there was no correction for multiple comparisons. The data on regional localization of effects of aging and dopamine blockade on network efficiency should therefore be regarded as exploratory in nature.

## Supporting Information

Figure S1 Giant Connected Cluster or Largest Subgraph Size as a Function of Cost for Young Brain Networks (Black Lines) and Old Brain Networks (Red Lines) Following Placebo (Left) and Sulpiride (Right) As expected, the percentage of regions connected to the largest subgraph increases to a maximum value of 100% as a monotonically increasing function of cost. In the small-world regime of costs, 0.05 ≤ *K* ≤ 0.34, the giant connected cluster or largest subgraph generally includes at least 80% of all possible regions.
(26 KB EPS)

Figure S2 Efficiency Measures Estimated and Integrated over the Small-World Regime of Costs, 0.05 ≤ *K* ≤ 0.34, for *Weighted* Networks (Rows 1,2) Global (left) and local (right) efficiency curves for young (black lines) and older (red lines) people after placebo (row 1) and sulpiride (row 2).
(Row 3) Within-subject effects of dopamine antagonism on integrated global efficiency (left) and integrated local efficiency (right): data on younger subjects are denoted by black lines and black triangles, data on older subjects by red lines and red triangles.
(Row 4) Box-plots showing median, interquartile range, and range for integrated global efficiency (left) and integrated local efficiency (right) in each age group after each treatment.
(52 KB EPS)

## Abbreviations

ANOVA: analysis of variance
BOLD: blood-oxygen-level-dependent
fMRI: functional magnetic resonance imaging
MRI: magnetic resonance imaging
OP: old people after placebo
OS: old people after sulpiride
YP: young people after placebo
YS: young people after sulpiride
